# Anti-gravity treadmill training benefits the post-operative rehabilitation of ACL reconstruction and the effects on the muscular atrophy and balance ability: a cohort study and 1y follow-up

**DOI:** 10.3389/fspor.2025.1654873

**Published:** 2025-09-29

**Authors:** Bing-Xian Ma, Yan-Song Qi, Zi-Heng Zhang, Ye Tian

**Affiliations:** ^1^ Department of Orthopedics, Shengjing Hospital of China Medical University, Shenyang, China; ^2^ Orthopedic Center (Sports Medicine Center), Inner Mongolia Autonomous Region People’s Hospital, Hohhot, China

**Keywords:** anti-gravity treadmill training, anterior cruciate ligament reconstruction, muscular atrophy, balance ability, post-operative rehabilitation, knee function, cohort study

## Abstract

**Background:**

Post-operative muscular atrophy and impaired balance are great rehabilitation challenges in patients with anterior cruciate ligament reconstruction (ACLR). Anti-gravity treadmill training (AGTT) is a novel rehabilitation therapy that provides partial body-weight support (BWS) and enhances muscular motor. The present study aims to explore the effects of AGTT rehabilitation on muscular atrophy and balance ability, as well as the outcomes of knee function, physical activity, and return to sports in ACLR patients.

**Methods:**

This was a prospective cohort study. ACLR patients were included between January 1, 2022, and December 31, 2023, and randomly divided into the AGTT group (6-week AGTT+routine rehabilitation protocol) and control group (routine rehabilitation protocol). After a 6-week AGTT, a muscular atrophy grading system was used to estimate the severity of quadriceps femoris atrophy, and the Berg balance scale (BBS) was used to evaluate the patients’ balance ability. At 1y follow-up, the outcome functional assessments were performed, including Lachman and pivot shift test, A-P ligament laxity (KT-2000), range of motion, International Knee Documentation Committee (IKDC) score, Tegner Activity Score (TAS), and rate of returning to sports.

**Results:**

73 ACLR patients have completed 1y follow-up, the total missing rate was 8.75%. After 6-week AGTT, the severity of quadriceps femoris atrophy was significantly decreased in the AGTT group (18/36, grade A/all) than the control group (7/37, *P* = 0.003), while the BBS in the AGTT group (53.67 ± 1.00) was significantly increased than the control group (52.12 ± 1.08, *P* < 0.001). At 1y follow-up, the A-P joint stability (22/36, normal/all), IKDC (81.61 ± 6.92), TAS (3.64 ± 0.76), and the rate of return to sports (26/36, yes/all) in AGTT group were significantly higher than the control group (10/37, *P* = 0.015) (78.63± 2.75, *P* = 0.020) (3.14± 0.75, *P* = 0.006) (16/37, *P* = 0.012).

**Conclusions:**

A 6-week AGTT rehabilitation can protect the quadriceps femoris muscular atrophy and promote balance recovery in ACLR patients, resulting in better short-term outcomes of joint stability, knee function, physical activity level, and return to sports.

**Level of evidence:**

Level 2.

## Introduction

Anterior cruciate ligament (ACL) is one of the main stabilizing structures of knee joint, ACL rupture can cause notable instability during motion, which usually needs ACL reconstruction (ACLR), especially in young and physically active populations. It has been generally considered that the post-operative rehabilitation of ACLR is essential, which is closely associated with the outcome of knee function, physical activity, time to return to sports, and quality of life (QoL).

Neuromuscular dysfunction related to muscular atrophy and impaired proprioceptive sensation is the greatest challenge during post-operative rehabilitation of ACLR (1–4), leading to muscle weakness, joint instability, and impaired static/dynamic balance ability (3), which strictly limit the knee functional recovery such as physical activity level and return to sports (4, 5). After ACL rupture, arthrogenic muscle inhibition (AMI) down-regulates motor-neuron excitability, which is known as the a critical factor that limits rehabilitation outcomes after ACLR (1). What's more, the remaining neuromuscular dysfunction post ACLR rehabilitation has been linked to increased risks of ACL re-rupture and post-traumatic knee osteoarthritis (KOA) (6). It has been reported that ACLR patients are often left with muscular atrophy and strength deficits of up to 40%, compared to the contralateral knee even after completing the rehabilitation period (7). The impaired proprioceptive sensation is inevitable because the initial trauma and potential surgical reconstruction cause the loss of proprioceptive neural receptors (8). Frontiers research in the field of ACLR rehabilitation should have an efficient intervention focusing on this neuromuscular dysfunction.

Anti-gravity treadmill training (AGTT) is a novel rehabilitation therapy for lower-extremity injuries, which uses positive air pressure to provide partial body-weight support (BWS) with 1% accuracy, thereby enhancing the lower-extremity muscular motor during rehabilitation tasks with less pain. It has been reported that AGTT can relieve pain and increase the muscle strength in KOA patients, thereby restoring community activities (9). Compared to the traditional rehabilitation protocols, AGTT increased the muscle activation and muscle mass in subjects with running injuries and promoted returning to sports (10). Randomized controlled trials (RCTs) have demonstrated that post-operative rehabilitation with AGTT can positively affect the lower-extremity dynamic balance in patients with lower-extremity fractures (11, 12). Considering AGTT can provide several benefits for muscle activation and balance ability (an important part of proprioceptive sensation) in the rehabilitation of those lower-extremity injuries, we hypothesize that AGTT rehabilitation may reverse AMI and promote quadriceps recovery in ACLR patients.

In 2024, a case report first reported the use of anti-gravity treadmill training on rehabilitation of ACL reconstruction in a professional soccer player, which highlights the movement strategy compensations during the rehabilitation (13). According to our knowledge, no systematic longitudinal clinical research has focused on the post-operative rehabilitation of ACLR using AGTT. Whether AGTT will have efficacy on post-operative rehabilitation of ACLR, as well as its impacts on muscular atrophy and proprioceptive sensations of the lower extremity remains unknown. Given that neuromuscular deficits increase the ACL re-rupture risk and correlate with early knee OA (6), demonstrating whether AGTT improves these long-term outcomes is highly relevant. We designed a prospective cohort study, aiming to explore the effects of AGTT rehabilitation on muscular atrophy and balance ability, as well as the outcomes of knee function, physical activity level, and return to sports in ACLR patients.

## Materials and methods

### Patient involvement

Patients who underwent arthroscopic ACLR were included between January 1, 2022, and December 31, 2023, in the Inner Mongolia Autonomous Region People's Hospital. The inclusion criteria were: (1) age: 18–50 years old, BMI ≤31; (2) unilateral ACL rupture, time from injury to ACLR ≤1 year; (3) ACLR with autologous double-bundle four-strand hamstring tendon; (4) agree to participate in this study after signing the informed consent; (5) agree to the arrangement of the rehabilitation subgrouping. The exclusion criteria were: (1) collateral ligament rupture or posterior ligament rupture; (2) ACL re-rupture; (3) history of lower-extremity fracture, ligament rupture, and operations of the bone-ligament system (14); (4) knee osteoarthritis with the Kellgren-Lawrence grade >2, rheumatoid arthritis, and gouty arthritis (14); (5) nerve system diseases.

Post-operative patients were randomly divided into the AGTT rehabilitation group and control group (routine rehabilitation scheme) with a ratio of 1:1. The study protocol was performed with the approval of the Ethics Committee of Inner Mongolia Autonomous Region People's Hospital. (Ethics Approval No. 202518406l).

### Rehabilitation protocols of the two groups

**Figure 1:**
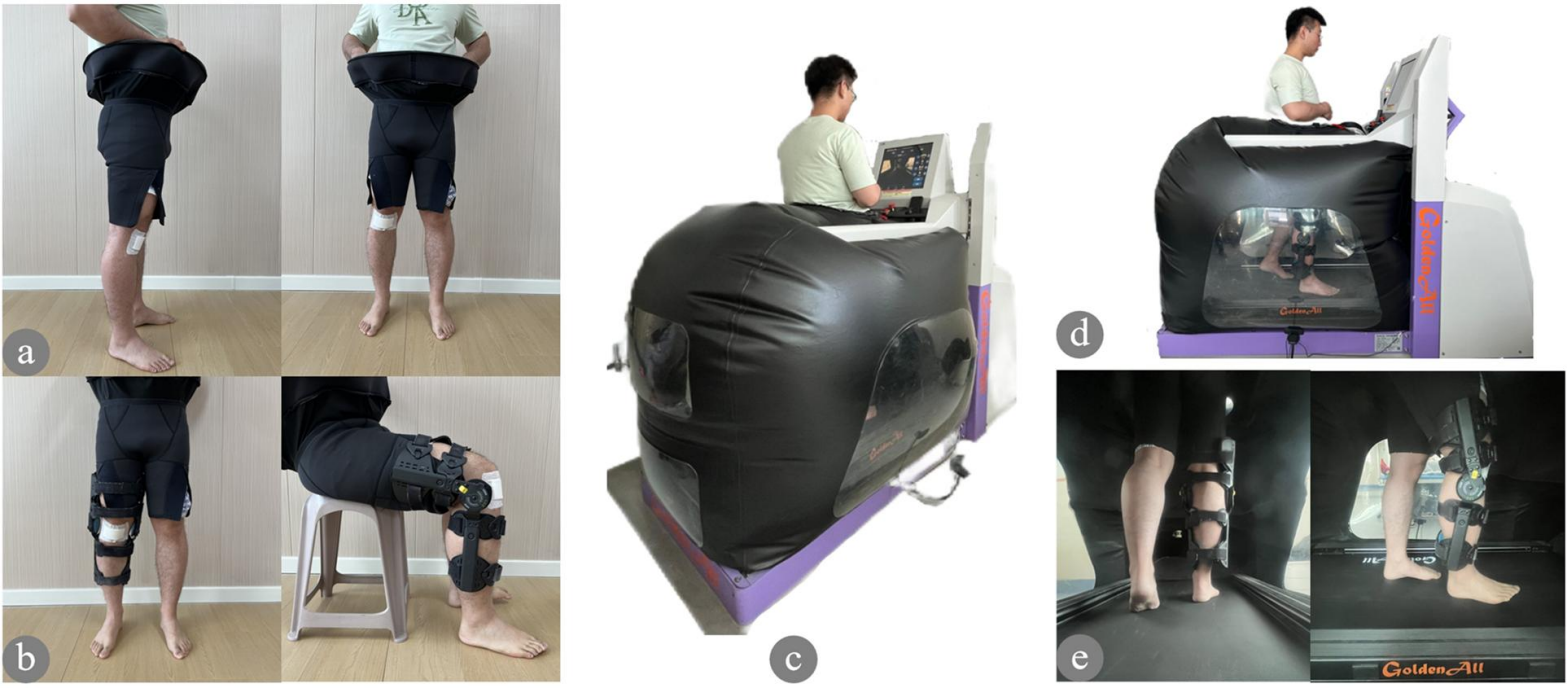
AGTT rehabilitation in post-operative ACLR patients; **(a)** patients wear the air-tight shorts after surgery; **(b)** wear the angle-adjustable knee brace; **(c)** AGTT walking task, the patient's lower extremity was airtightly packaged in the pneumatic chamber with a positive air pressure; **(d)** Side view of the pneumatic chamber; **(e)** internal monitoring view of the pneumatic chamber.

AGTT was performed by an AGTT device (GA100S, Golden All, China) (Figure 1). The AGTT rehabilitation was started from the 3rd week after surgery and was conducted 3 times/week for a total duration of 6 weeks (18 sessions for each participant, 30 min/session). AGTT rehabilitation protocols: (1) the initial 2 weeks of AGTT: walking for 30 min (speed = 0.5 km/h, BW = 20%, incline = 0%); (2) 3–4 weeks of AGTT: walking for 30 min [speed = 0.6–0.8 km/h (15), BW = 20%, incline = 0%]; (3) 5–6 weeks of AGTT: walking for 30 min [speed = 0.8–0.9 km/h (15), BW = 20%, incline = 1%–2% (15)]. To ensure the safety and efficacy during AGTT rehabilitation, one senior physiotherapist was assigned to supervise and manage the rehabilitation (Table 1).

**Table 1:** The AGTT rehabilitation protocol for ACLR patients in post-operation.

Time (w)	Speed (km/h)	Body-weight support (%)	Incline (°)	Duration of training (min)
1–2 (3rd–4th week after ACLR)	0.5	20	0	30
3–4 (5th–6th week after ACLR)	0.6–0.8	20	0	30
5–6 (7th–8th week after ACLR)	0.8–0.9	20	0	30

Both the AGTT rehabilitation group and control group underwent the same routine rehabilitation scheme: (1) immediate post-operative phase: the isometric contraction of the quadriceps was started as soon as possible after surgery (14); (2) early post-operative phase (the first 2 weeks): a lockable functional brace (0°) was used for a whole day, a ROM of 90° should be achieved during this period; (3) intermediate post-operative phase (3–4 weeks): a lockable functional brace (0°–30°) was used during walking for the next 2 weeks, a ROM of 100° should be achieved; (4) late post-operative (5–8 weeks): a lockable functional brace (0°–90°) was used during walking for the next 2 weeks, a ROM of 130° should be achieved; (5) the transitional phase (9 weeks–6 months): exercise training and return to sports.

### Rehabilitation assessment on muscular atrophy

Muscular atrophy was estimated by measuring the differences of thigh circumference between the ACLR side and the contralateral side, at 10 cm above the upper border of the patella. The muscular atrophy was graded as follows: grade A (no difference), grade B (0–1 cm), grade C (1–2 cm), and grade D (>2 cm) (16). Rehabilitation assessment on muscular atrophy was performed at the end of the 8th week after ACLR (AGTT rehabilitation was also completed in the 8th week).

### Rehabilitation assessment on balance ability

Berg balance scale (BBS) was used to evaluate balance ability, consisting of 14 simple functionally oriented balance tasks, beginning with “standing up” and progressing up to “stand upright without support”, “stand upright with closed eyes”, and “standing on one leg” etc. (17). Each task was scored by a five-point scale (0–4), and a total score between 0 and 56 was generated by summing those scores together at the end of the BBS test, a higher score indicated a better balance ability. The rehabilitation assessment on balance ability was performed at the end of the 8th week after ACLR.

### Follow-ups

The follow-up was started when the ACLR operation was completed. The end was ACL re-rupture/severe complication/death/missing/1 year (y) post operation, whichever occurred first. General clinical parameters included: age, gender, body mass index (BMI), whether combined with a meniscus tear, the graft diameter of ACLR, follow-up time, and complications.

The clinical assessments were performed at the end of the follow-up, including: the physical examination of ACL stability, ligament laxity (KT-2000), and knee range of motion (ROM), as well as the subjective scoring systems of knee function and physical activity. Outcome assessors were blinded to group assignment.

### Clinical examinations of knee joint

The clinical examinations of knee stability were performed 1y post-operatively in all ACLR patients, consisting of the Lachman test and pivot shift test, which are generally used to determine the knee stability recovery after ACLR (18). Lachman test was used to assess the A-P joint stability, classified as: hard end-point (−), doubtable laxity (±), and soft end-point (+) (14). Pivot shift was used to assess the rotational stability, classified as: normal (−), glide (±), and clunk or gross (+) (19).

A-P ligament laxity was measured by KT-2000 when the knee is flexed at 30°, by comparing it to the contralateral healthy side, classified as: normal, grade 1 (difference between 1 and 5 mm), grade 2 (between 5 and 10 mm), and grade 3 (>10 mm) (19).

Knee range of motion (ROM) was measured by standardized goniometry technique in pre-operation and at 1y follow-up.

### Subjective assessments of knee function

The International Knee Documentation Committee (IKDC) subjective-form score and Tegner Activity Score (TAS) were used to assess the outcome of knee function and physical activity correspondingly, at 1y follow-up (20, 21). IKDC scoring was performed by self-questionnaires with a full score of 100, and a higher score represented a better functional outcome. TAS was also performed by self-questionnaires, and a score of 10 was assigned based on the level of activity that the patient selected as best representing their current activity level, for example, a score of 0 represented “sick leave or disability pension because of knee problems”, while a score of 10 corresponded to “participation in national and international elite competitive sports” (21).

The rate of returning to sports at 1y follow-up was also used to evaluate the outcome of physical activity.

### Statistical analysis

All of the statistical analyses were performed using SPSS 20.0 (SPSS Inc., 2009, Chicago, IL, USA). The continuous data were expressed as mean ± SD, and the count data (gender, combined with/without meniscus tear, muscular atrophy grading, complications, Lachman test result, Pivot shift result, A-P Laxity grading, and return to sports rate) were expressed as number (*n*) and rate (/). All of the continuous data in the present study were tested by the Shapiro–Wilk normality test. Intra-group comparisons of the continuous data were processed by the independent samples *t*-tests and Levene variance homogeneity tests between groups, while intra-group comparisons of the count data were processed by the Chi-square test or Fisher's exact test. The level of significance was set at 0.05.

## Results

### Basic characteristics

Initially, 80 ACLR patients were continuously included in this prospective cohort study and completed the rehabilitation task, by a ratio of 1:1 in the AGTT group and control group. At 1y follow-up, 2 patients were missing (can not be reached in any way) and 2 patients declaimed that he/she was not living in the city during the follow-up time in the AGTT group, 1 patient was missing and 1 patient declaimed that he moved to another city, and 1 patient died of pancreatic cancer in the control group. Finally, 73 ACLR patients have completed 1y follow-up, the total missing rate was 8.75% (AGTT 4/36, Control 3/37), and the minimal follow-up was 12 months.

The basic characteristics of the AGTT group and control group were listed below, no significant difference of gender, age, BMI, whether combined with meniscus tear, graft diameter, and follow-up time was observed between the 2 groups (Table 2).

**Table 2:** Basic characteristics of ACLR patients in the AGTT and control groups.

Basic characteristics		AGTT (*n* = 36)	Control (*n* = 37)	*P*-value
Gender	Male	24	22	χ^2^ = 0.407 *P* = 0.524
	Female	12	15	
Age (year)		33.9 ± 6.8	33.7 ± 9.0	*t* = 0.130 *P* = 0.897
BMI		24.27 ± 2.03	24.35 ± 1.13	*t* = −0.220 *P* = 0.827
Meniscus tear	With	17	16	χ^2^ = 0.117 *P* = 0.733
	Without	19	21	
Graft diameter (mm)		7.85 ± 0.23	7.78 ± 0.45	*t* = 0.760 *P* = 0.451
Follow-up time (mon)		13.1 ± 1.0	12.9 ± 1.0	*t* = 0.362 *P* = 0.488

The age, BMI, and follow-up time were normally distributed and tested by the Shapiro–Wilk normality test.

### Rehabilitation assessment on neuromuscular function

At the end of the 8th week after ACLR (AGTT rehabilitation was also completed at the 8th week), the rate of grade A of muscular atrophy grading system in the AGTT group was significantly higher than the control group (50.0% vs. 18.9%, *P* = 0.003) (Table 3), which also means the rate of grade B, C, D in the control group was significantly higher than the AGTT group (Table 3). The BBS of the AGTT group was significantly higher than that of the control group with the difference of 1.55 points (95% CI: 1.05–2.05) (Table 3).

**Table 3:** Neuromuscular parameters in the AGTT group and control group after ACLR rehabilitation.

Neuromuscular parameters		AGTT (*n* = 36)	Control (*n* = 37)	*P*-value
Muscular atrophy	Grade A	18	7	χ^2^ = 13.899 *P* = 0.003*
	Grade B	14	12	
	Grade C	3	13	
	Grade D	1	5	
BBS (point)		53.67 ± 1.00	52.12 ± 1.08	*t* = 6.349 *P* < 0.001*
Complications	With	5	8	χ^2^ = 0.745 *P* = 0.388
	Without	31	29	

Muscular atrophy was graded as follows: grade A (no difference), grade B (0–1 cm), grade C (1–2 cm), and grade D (>2 cm); the BBS was normally distributed and tested by the Shapiro–Wilk normality test.

* *P* < 0.01.

5 patients had complications during the AGTT rehabilitation period: 3 patients had transient edema, and 2 patients had transient pain (mild) of the knee joint. 8 patients had complications during the rehabilitation period in the control group: 4 patients had transient edema, 2 patients had transient pain (mild) of the knee joint, and 2 patients had experienced limited ROM (intervened by a rehabilitation therapist using manual release). All those complications were relieved or disappeared in 1 week. However, no significant difference of the complication rate was observed between the 2 groups (Table 3).

### Clinical assessments on functional outcomes

At 1y follow-up, the rate of normal A-P laxity, IKDC, TAS and rate of return-to-sport in the AGTT group were significantly higher than that in the control group (Table 4). AGTT improved return-to-sport by 29 percentage points (72.2% vs. 43.2%, *P* = 0.012) than the control group (Table 4). The Lachman test, pivot shift test, and ROM did not show a significant difference between the 2 groups (Table 4).

**Table 4:** Outcome knee function assessments of AGTT group and control group at 1y follow-up.

Parameters		AGTT (*n* = 36)	Control (*n* = 37)	*P*-value
Lachman	−	30	24	χ^2^ = 3.533 *P* = 0.139
	±	6	12	
	+	0	1	
Pivot shift	−	30	28	χ^2^ = 1.294 *P* = 0.664
	±	6	8	
	+	0	1	
A-P laxity	Normal	22	10	χ^2^ = 9.286 *P* = 0.015*
	Grade 1	11	18	
	Grade 2	3	7	
	Grade 3	0	2	
ROM (°)		132.07 ± 4.96	131.25 ± 5.73	*t* = 0.654 *P* = 0.515
IKDC (point)		81.61 ± 6.92	78.63 ± 2.75	*t* = 2.402 *P* = 0.020*
TAS (point)		3.64 ± 0.76	3.14 ± 0.75	*t* = 2.845 *P* = 0.006**
Return to sports at 1y	Yes	26	16	χ^2^ = 6.272 *P* = 0.012*
	No	10	21	

The Lachman test result was classified as: hard end-point (−), doubtable laxity (±), and soft end-point (+); Pivot shift result classified as: normal (−), glide (±), and clunk or gross (+); A-P ligament laxity was classified as: normal, grade 1 (difference between 1 and 5 mm), grade 2 (between 5 and 10 mm), and grade 3 (>10 mm); the ROM, IKDC, and TAS was normally distributed and tested by the Shapiro–Wilk normality test.

* *P* < 0.05.

** *P* < 0.01.

## Discussion

### AGTT promotes the recovery of neuromuscular function in ACLR patients

The present study found that a 6-week AGTT rehabilitation can significantly decrease the severity of muscular atrophy of the affected lower extremity and increase the BBS of ACLR patients in the AGTT group, compared to the control group, suggesting AGTT rehabilitation can promote the recovery of neuromuscular function at the post-operative phase of ACLR. Although the promoting effects of AGTT on lower-extremity recovery in post-operative phases have been reported in many kinds of surgeries, such as TKA (22) and lower-extremity fracture fixations (11, 12), no systematic longitudinal cohort study or RCTs has focused on its clinical application in ACLR patients, according to our knowledge. The present study firstly reports the efficacy of AGTT rehabilitation on the muscular atrophy and impaired balance ability in the post-operative phase of ACLR patients.

Muscular atrophy on the ACLR side is one of the biggest problems during the post-operative rehabilitation (1–4). An ACL rupture should not be considered as a “simple” musculoskeletal pathology with local mechanical and motor dysfunctions (2), together with the limited physical level in post-operation and the denervation of musculature caused by ACL rupture, contributing notable post-operative muscular atrophy. The potential mechanisms associated with the muscular atrophy in ACLR patients include: (1) disuse-related anabolic resistance, resulting in an increased muscle breakdown and decreased muscle synthesis (5, 23); (2) decreased muscle remodeling following ACL rupture (5, 24); (3) neuromuscular changes resulting from the decrease in the number of neuromuscular junctions (denervation of musculature) (5, 25). Hence, the muscular atrophy in ACLR patients is considered and identified as unresponsive to a traditional rehabilitation protocol, such as strength training (5). Muscular atrophy of the lower extremity in ACLR patients usually occurs in the quadriceps femoris, which is the most main knee extensor, leading to a significantly reduced muscular strength of knee extension and joint stability. It has been reported that after traditional rehabilitation, ACLR patients can be left with quadriceps femoris atrophy and strength deficits of up to 40% compared to the contralateral knee (7). The AGTT protocol in the present study restricted the BWS to 20%, which enhanced the lower-extremity muscular exercising in the early rehabilitation phase with a much lower load, hence may protect the muscular mass from disuse-related anabolic resistance. It has been reported that AGTT can lower the impact forces and metabolic demand of exercising (10), helping to maintain the metabolic demand and fitness while minimizing bone and tissue loading. Liang et al. have found that AGTT can improve the muscle strength values of the rectus femoris, semitendinosus, and biceps femoris in KOA patients (9). What's more, we found that AGTT can also promote the recovery of proprioceptive sensation, which may contribute to muscular remodeling and help to protect the musculature denervation in ACLR patients. Enhanced muscular exercising under the restricted BWS by AGTT starting from the early rehabilitation phase, contributes to the muscular remodeling and helps to protect the musculature denervation, which should be the potential reason for the lower severity of quadriceps femoris atrophy compared to the routine rehabilitation protocol in the present study.

Balance is an important part of proprioceptive sensation. Due to the loss of proprioceptive neural receptors (denervation) caused by the initial trauma and the following ACLR (8), impaired balance ability is inevitable in every patient. It has been found that ACLR patients have lesser bilateral corticospinal excitability than those without injury (26), and the initial trauma and the following ACLR caused loss of Golgi tendon organ-like receptors, contributes to the dysfunction of information input to the central nervous system (CNS), which is responsible for the impaired static/dynamic balance and motor control (8). BBS has been reported to be a reliable and valid tool to assess balance in a variety of patient populations such as KOA, it has been reported that BBS has a high validity (ICC = 0.95) in KOA patients (27). The present result indicated that AGTT rehabilitation in post-operation of ACLR can promote the recovery of balance ability. It has been reported that AGTT can stimulate the sensitivity of the central motor nerve pathway, and improve the balance ability and gait in stroke patients through the enhancement of proprioceptive input to the CNS in stroke patients (28, 29). In general consensus, the post-operative rehabilitation period consists of the following 5 phases: an immediate post-operative phase, early and intermediate post-operative phases (these 3 stages usually have a marked neuromuscular dysfunction), the late post-operative and the transitional phases (contain more tasks such as running and jumping) (2). The AGTT protocol in the present study consisted of different speed and incline modes at different rehabilitation phases, starting from the intermediate post-operative phase (3–4 weeks) to the late post-operative phase (5–8 weeks), with the speed starting from 0.5 km/h to 0.8–0.9 km/h (15), the incline starting from 0% to 2% (15). The initial 2 weeks were a practice period, which can ensure patients’ safety and adaptation to the BW and speed set, as well as the following incline set. There is evidence suggesting that the variation in speed and incline of AGTT protocol can introduce a dynamic challenge to the patients' balance ability and motor control, which is essential for retraining the neural pathways of proprioception sense (30, 31). It has been demonstrated that both the speed and incline variation in an AGTT protocol are beneficial for the improvements in knee extensor muscle strength and balance, suggesting an AGTT protocol with different speed and incline modes can promote the recovery of impaired proprioception sense that a standard AGTT does not (15). We considered that the 6-week AGTT protocol with variations in speed and incline starting from the 3rd week in post-operation of ACLR may enhance the neural pathway of proprioceptive sense, thus promoting the patients' balance ability, which may explain the reason why the AGTT group had a higher BBS compared with the control group.

### AGTT promotes the outcome of knee function in ACLR patients

The present study found that the A-P joint stability, IKDC, TAS, and the rate of return to sports in the AGTT group were significantly higher than that in the control group at 1y follow-up, suggesting AGTT rehabilitation can promote the short-come outcome of knee function in ACLR patients. Musculature strength and activity is one of the main stabilizers of joint, it has been demonstrated that muscular activity function was an independent factor associated with joint stability (32, 33). As the quadriceps femoris is the strongest musculature of knee, we considered that the efficacy of AGTT on the muscular atrophy was the main potential reason for the better A-P joint stability at 1y follow-up, compared to the routine rehabilitation protocol.

TAS provides a standardized assessment system for grading the outcome of physical activity level, remedying the limitation of lacking physical activity level in some knee function subjective assessment systems, such as Lysholm and IKDC (21). The present result suggested that a 6-week AGTT rehabilitation can significantly improve the knee functional outcomes in ACLR patients at 1y follow-up, compared to a routine rehabilitation protocol, especially the functional outcomes on physical activity level and return to sports. We considered that the efficacy of AGTT rehabilitation on muscular atrophy and balance ability contributed to the higher IKDC, TAS, and rate of return to sports in the AGTT group. It has been reported that quadriceps femoris muscular atrophy can subsequently impact the success of rehabilitation and patients' daily function (5), while an impaired balance ability can result in impaired motor control, gait disturbance, and decreased daily living activity level (34). Impaired static/dynamic balance ability in ACLR patients can strictly limit knee function recovery, such as physical activity level and return to sports (4, 5). Similar to our results, Liang et al. found that AGTT rehabilitation can improve the muscle strength of rectus femoris, semitendinosus, and biceps femoris, thus improving 10-m walking test and Timed-up-and-go test results and community activity level in KOA patients (9). We considered that the AGTT protocol with variations in speed and incline may be the potential reason explaining the better functional outcomes in the AGTT group. It has been demonstrated that both the speed and incline variation in an AGTT task benefited the improvements in knee muscular strength and balance ability, as well as the activities of daily living (ADLs) in stroke patients, suggesting an AGTT protocol with different speed and incline modes can promote the functional outcomes that a standard AGTT does not (15). The present results indicated that the AGTT protocol with variations in speed and incline can protect the muscular atrophy and promote the recovery of balance ability, thereby benefiting the functional outcomes of physical activity and return to sports in ACLR patients.

### Limitations

Our study had several limitations. First, it was a pilot study, which had a relatively small sample size and a short-term follow-up result. The sample size was determined by single-center annual surgical volume and feasibility, this is an exploratory study that warrants replication in larger samples. Longitudinal RCTs and cohort studies with more samples and longer follow-up times are needed to determine the long-term functional outcomes of AGTT rehabilitation in ACLR patients. Second, we did not record the baseline physical activity level, which may have a potential effect on results. Besides, we only detected the functional test parameter of balance ability, neurophysiological tests of the corticospinal excitability and neural pathways are still needed to further prove the potential mechanism of AGTT on proprioception sense.

### Conclusions

The present cohort study found that the AGTT rehabilitation starting from the 3rd week in post-operation can protect quadriceps femoris muscular atrophy and promote balance recovery in ACLR patients, resulting in better short-term outcomes of joint stability, knee function, physical activity level, and return to sports. The variations in speed and incline of AGTT protocol may be the potential reason for the better efficacy and outcomes compared to the routine rehabilitation protocol.

## Data Availability

The original contributions presented in the study are included in the article/Supplementary Material, further inquiries can be directed to the corresponding author.
